# Values-Based Foundation for a U.S. Single Payer Health System Model

**DOI:** 10.3389/fsoc.2021.627560

**Published:** 2021-04-28

**Authors:** Walter Markowitz, Renee McLeod-Sordjan

**Affiliations:** School of Health Professions, School of Nursing, Hofstra University, Hempstead, NY, United States

**Keywords:** single payer health care, value based ethics, single payer health care reform, Kantian ethics, health care for all

## Abstract

A universal, single payer model for the American health system aligns with and should emanate from commonly held values contained within the country’s foundational religious teachings, morals, ethics and democratic heritage. The Affordable Care Act in its attempt to create expanded health access has met with significant challenges. The conservative Supreme Court decreases the likelihood of a federal mandated single payer model. As uncertainty of the structure of the healthcare system increases, this paper supports its transformation to a single payer model. Healthcare should be considered a duty within the framework of a Kantian approach to ethics and a social good. Evidently ignoring this duty, the American health system perpetuates a healthcare underclass, with underserved portions of the population, with unequal access to quality care and persistent health status and outcome disparities. The COVID-19 pandemic demonstrated the effect of social determinants on optimal health outcome. A health insurance system based on the nation’s commonly held values has the potential to eliminate these disparities.

## Introduction

A health system can be compared to a symphony, in which all musicians harmoniously work together to a common goal. By contrast, the United States (U.S.) health system is more like a cacophony of sounds. Discordance emanates from its pluralism of for-profit, not-for profit, faith-based and municipal providers, regulators and payers. Each competes to benefit respective positions rather than common goals. It has resulted in comparatively more expense, with poorer and disparate population health status and outcomes. Even with the changes brought about by the Affordable Care Act [ACA], approximately one in five privately insured individuals say they skip needed care because of cost, while larger shares of Americans particularly those with high deductible plans have experienced some form of financial strain paying for care. More than 70% of Americans say that the U.S. healthcare e system needs either “fundamental changes” or to be “completely rebuilt.” (Mound, 2018).

With a single payer model all have the same access to the same services and providers are paid the same for the same service. Premiums to insurance companies would be replaced by a taxing system (Seidman, 2015). The model fulfills the World Health Organization’s Declaration of Alma, challenging society to provide health care as a right (World Health Organization, 1978).

## Disparities Within the Current U.S. Health System

The elimination of health disparities and the achievement of health equity appears as an overarching goal within the framework of the latest iteration of the Department of Health Services’ *Healthy People* (2030) (United States Department of Health and Human Services, 2020a). This has appeared as an overarching goal within multiple *Healthy People* decennial iterations (United States Department of Health and Human Services, 1996; United States Department of Health and Human Services, 2015; United States Department of Health and Human Services, 2020b). Despite the inclusion of the elimination of health disparities and the achievement of health equity as an overarching goal for the nation for decades, health disparities remain all too evident and in some instances have grown even larger. Currently still, there is a health care underclass where lower income groups and racial/ethnic minorities do not have equal access to care (Artiga et al., 2020), with resultant evident disparities related to such health status indicators, as life expectancy at birth, infant mortality and preterm births (National Center for Health Statistics, 2016). Non-Hispanic Caucasian women have the lowest infant mortality rate of 4.63 (per 1,000 live births), compared to 4.86 Hispanics, and 10.75 for non-Hispanic Black women.

Controlling for socioeconomic status (SES) there are evident disparities by race and ethnicity. Even when other factors are comparable, marginalized racial and ethnic populations tend to receive care that is of lower quality. Non-Hispanic Black males have the highest cancer mortality rate, 16% higher than Non-Hispanic Whites (NHW) and double that of male Asian or Pacific Islanders. Black males’ prostate cancer mortality rate is more than twice that of the other racial/ethnic groups. Black females have a breast cancer mortality rate 40% higher than NHW females, although their rates of incidence are similar for the two groups.

Life expectancy disparities based on income are striking. At the age of forty (40), men whose income was at the lowest 1% level had an expected death age of 72.7 years. Men at the highest 1% income level had an expected death age nearly fifteen (15) years greater (87.3 years). For women at the age of forty (40) at the lowest compared to the highest income levels, the difference was approximately ten (10) years, i.e. 78.8 and 88.9 respectively (Chetty et al., 2016).

Fiscella and Sanders found the uninsured have much lower rates of receiving preventive care. African Americans and Latinos have lower rates of cancer screening, most evident with the uninsured (Fiscella and Sanders, 2016). “Significant disparities by race and ethnicity are seen in quality of care for chronic disease control.” This includes poorer control of blood pressure, blood sugar and LDL cholesterol levels. Minority patients are hospitalized and re-hospitalized at higher rates. African Americans and Latinos use mental health and substance abuse services far less than Whites do.

Powell (Powell, 2016) asserted that it was extremely difficult to disentangle health inequality from so many other barrier-creating social determinants, such as income, education, housing and geography, as well as immutable factors such as race and gender. In many instances establishing cause vs. effect is likewise difficult to discern. In 2003, the Institute of Medicine (IOM) highlighted social inequity and lower quality of care experienced by racial and ethnically diverse individuals, even when access-related factors, such as patients’ insurance status and income, are controlled. Moreover, health systems payer models, as well as the legal regulatory, and policy environment in which they operate, may have disparate and negative effects on minorities’ ability to attain quality care (Institute of Medicine, 2003).

Inconsistent ACA implementation among states has perpetuated disparate access to health insurance. In 2012, the U.S. Supreme Court ruled each state could determine whether they would expand Medicaid financial eligibility for its citizens from those earning at or below the federal poverty level up to 138% of the federal poverty level. Following the ruling fourteen states opted out of the financial eligibility expansion. It was estimated as result of that decision, that 3.6 million fewer people would be covered by Medicaid. It was further estimated that states could lose $8.4 billion in federal transfer payments and state spending for uncompensated care could increase by $1 billion in the ensuing four years (Price and Eibner, 2013). The inconsistent implementation of ACA created what some have labeled a coverage gap in states which opted not to expand Medicaid financial eligibility up to 138% of the federal poverty level. Resultant uninsured populations were concentrated in the southern states of Texas, Florida and Georgia, with 25%, 18% and 10% respectively. Hispanics/Latinos have an uninsured rate that is three times that of Whites and for Blacks the rate is double the White rate (Texas Health Institute, 2016).

## Comparable Single Payer Health Systems

There is marked heterogeneity among single payer health system models. Denmark, Sweden, Australia, England, France, Germany, the Netherlands, Norway, Singapore, Switzerland, Taiwan and Canada are example of 12 high income countries with single payer financing of health care. Countries vary in terms of the extent to which regional or national government exert financial and regulatory control. They also differ in terms of the scope of health coverage, hospital ownership, innovative technological adoption, budgetary regulationsand degree of financial cost to the insured.

Managing healthcare exclusively at a federal level, such as Medicare, without regional control (Medicaid) is a rarity seen only in the Netherlands, France, Singapore and Taiwan. Out of pocket expenditures are highest among federal single payer models. For example, in Singapore, 69% of constituents have private health insurance and 61% of total health expenditures are paid by consumers. One may argue with the exception of France, these countries do not compare in size to the American Health System (Glied et al., 2019). When looking at France only 7% of the total health expenditures are paid by consumers but 95% of the population has private insurance. France spends less than half of per capita expenditures than the United States. Life expectancy in France is four years higher (78 years vs. 82); rehospitalization rates over 65 is 5% lower (14.7 vs. 20); infant mortality is lower (3.5 vs. 5.7%). The French system is government financed not government administered and given to its residents at birth. Called “social security” its focus is preventative care.

In countries where regional governments administrate health care under national policy, the percentage of out of pocket expenditures is twice the rate of France at about 14%–15%. Canada, Germany, Sweden and Switzerland apply this model. The rate of private health insurance range from 10% to 29% in these models except for Canada. Sixty-seven percent of Canadians have private health insurance. The Canadian system has a narrow set of basic federal benefits with comprehensive care covered by the regional provinces. The Canadian system has no cost sharing to the consumer. The Canadian model approximates Medicare and Medicaid for all.

With the exception of Taiwan, the high-income countries with moderate cost sharing have embraced UHC (UHC) for its population with at least a significant portion of the population purchasing supplemental private insurance to pay for uncovered services.

Systems of universal coverage vary, using a combination of taxes, premium payments and cost sharing. Almost all have a role for the private health insurance sector (Tikkanen, 2019). In contrast, the United States, spent 17.0 percent of its Gross Domestic Product (GDP) on health care. This spending represents almost twice the average among the 12 nations listed, with the poorest health outcomes including lowest life expectancy, highest suicide rate, highest prevalence of chronic diseases, highest number of preventable hospitalizations and highest rate of avoidable deaths (Tikkanen and Abrams, 2020).

In countries with cost sharing the United States still demonstrates poor health status indicators related to expenditures. Life expectancy in years is lowest (Switzerland, 83.6, Norway, 82.7 years; Canada, 82.0; U.S. 78.6.). Suicide rates is highest per 100,000 population (U.S. 13.9; Canada, 11.8; Norway, 11.6; Switzerland, 11.2). Chronic disease burden percentage in the population is highest (U.S: 28%; Canada, 22%; Norway, 16%; Switzerland, 15%) Avoidable death rates per 100,000 population is highest (U.S. 112; Germany, 86; Canada, 72; Switzerland 54).

### Pandemic & Universal Health Coverage

A study during the COVID-19 pandemic has shown that countries with universal health coverage (UHC) had a case fatality rate of 10.5% compared to 4.9% for countries without UHC (Lee et al., 2021). Although these statistics were stark, in the initial months of the COVID-19 pandemic the results were attributed to prolonged wait times and allocation of life sustaining treatments to health care professionals. The fatality rate belies the fact that countries with UHC had lower case numbers of patients.

Recent literature illustrates the public health benefit of UHC to primary care; particularly vaccination. Dongawar and colleague (Dongarwar and Salihu, 2021) illustrated that among 47 countries that initiated COVID-19 vaccination by January 2021 more than half had UHC with a statistically significant (*p*-value < 0.5) early vaccination rate of 1.55% for nations with UHC vs. 0.51% for nations without UHC. An uncoordinated effort in the U.S. led to a vaccination rate of 2.82% which when compared to Denmark at 2.02% was higher but Israel had the highest vaccination rate of greater than 22%. Isreal’s health expenditures are also only 7% of GDP more than 50% less than the American percentage of GDP (Clarfield et al., 2017). Their UHC is funded through taxes and as the other aforementioned UHC health systems have public options with supplementary private coverage. Although the size of the nation is comparable to New York State, hospitals remain government owned and costs are constrained by governmental control.

Taiwan in particular demonstrated a profound proactive preventive approach to COVID-19. Taiwan with an increased population density and close proximity to Wuhan China experienced an incident rate of 20.7 cases per million compared to just New York State alone at 39.1 cases per million in april 2020. Perhaps the message of providing for public health led to Taiwan’s strategic priorities during the COVID-19 pandemic which included national public health agencies, investing in infrastructure and improving public health workforce. In the United States, in the midst of the COVID-19 pandemic health outcomes diminished for those with co-morbid and underlying conditions without health insurance. Despite the ACA, an estimated additional 5.4 million Americans lacked health insurance due to loss of employment during COVID-19. Medicare/Medical for All seeks to expand public benefits with suggestion of the elimination of private payors. Yet as discussed, comparable health systems with federally mandated systems expand access to all through supplementary private health insurance and cost-sharing. To strategically improve the American health system, foundational ethical and moral philosophies have implications to aid the adoption of universal health care.

## Ethical/Moral Values as Foundational for the Health System

Immanuel Kant’s categorical imperative includes two types of duties within his ethical and moral philosophy. There are positive duties, which include actions we are commanded to take and there are negative actions which are prohibited. Kant assumes that people are rational and have choices, which selected are to be based on rationality and duty (Yudanin, 2015). “The primacy of duty is affirmed in Kantian ethics. In true sense the moral worth of a person is revealed only when he acts from duty. Actions qualify as moral when they are worthy and enacted upon for the sake of duty (Mulia et al., 2016). Actions should be taken because they are inherently good onto themselves and not a means to achieve something else (Foot, 1972). Promoting access and health equality can be viewed as a positive duty, a moral action, a good onto itself within Kant’s categorical imperative.

According to a deontological philosophy actions are morally acceptable when consistent with relevant moral norms. In the case of universal health care in America, strategically adopting the norms of health systems with equitable health outcomes should be the duty of legislators. What should serve as the moral norms; what is right and what is wrong; what is a duty and obligation? Ross’ duties for pluralistic deontology assists in answering these questions. Consider their connectivity to foundational values:

1) Duties deriving from our own previous acts or actions: a) keeping promises, be they explicit or implicit.
3) Duties of justice … they guarantee that people can get what they deserve.
4) Duties of beneficence, which rest on the mere fact that there are other beings in the world whose condition we can make better in respect of virtue, or of intelligence, or of pleasure …
6) Non-maleficence, ensuring that no harm occurs to the ill, the infirmed the disenfranchized (Craig, 2014)

Craig (King, 2006) considered health care to be a social good, based on the tenets of religion, American ideals, morality and ethics for the foundations for the health system. The author challenges Americans to get away from looking in the mirror as the wicked witch did in *Snow White.* Americans are really not the “fairest of them all.” In looking in the mirror Americans must evaluate who we really are as a society what we should be, using our values to provide directionality as we struggle to provide a more rational, a more just health system. Dr Martin Luther King Jr reminded the nation, soon after the 1964 Civil Rights Law and the passage of Medicare and Medicaid, there was more to be done when he proclaimed: “Of all the forms of inequality, injustice in health care is the most shocking and inhumane.” (Meadowcroft, 2015) The provision of healthcare as a means of providing life, liberty and access to should not be determined by market forces.

## Duty, Morality and Commitment to Others

Friedberg (Friedberg, 2013) points to the Jewish philosopher Maimonides who wrote about the mitsvat aseh, representing an absolute obligation. The term mitzvah refers to such an obligation or commandment in Hebrew writings. While we are commanded or are obliged to perform mitzvot, when done we are blessed. Performing mitzvot provides the performer with recompense which should not be viewed as monetary reward. Biblical references to the blessings that will accrue if mitzvot are performed can be found for example in Leviticus 26: 3–12; Deuteronomy 7: 12–24; Deuteronomy11: 22–25; and Matthew 7: 24.

Tzedakah, is a related Hebrew term for the commandment associated with charity, which has the literal meaning of righteousness or justice. Consider the following capturing the essence of this mitzvah of tzedakah from Rabbenu Bachya Ben Asher, a 13th century Torah commentator:

“justice shall be pursued whether to one's profit or loss, whether in words or an action, whether to Jews or non-Jews. Hence we are not to wait for the right opportunity, the right time, and the right place to come along, but instead we are to actively seek the opportunity to practice justice. As a matter of simple justice, we are duty-bound to help others in need”. (Taitz, 2007).

## Complementary Values Support a Single Payer Model

A review of common ethical, moral and religious teachings, foundational to the nation’s heritage appear to support a single payer model. Inherent in such a model are values contained within the Golden Rule, a sense of community and responsibilities for those within the community, a responsibility to help those in need, compassion, justice and doing the right things. appropriately labeled health care as a social good.

A single payer system embodies these values. Its success is dependent on the public’s acceptance of two complementary principles: “1) subsidies for individuals who are too poor or too sick to acquire insuranc, and 2) compulsion (i.e. a mandate) for everyone else to participate and implicitly contribute to the subsidies. The United States could achieve universal coverage relatively promptly if it were willing to adopt these 2 principles.” (Fuchs, 2018) The two principles are evidently compatible with religious, ethical, and moral tenets.

## Barriers and Opposition to Policy Change

Unfortunately, as political polarity is reality, opposing sides ascribe mean-spirited attributes to their opponents. The following quote exemplifies this sentiment; “Some liberals presume that the sole motivation behind conservative resistance to UHC is crass selfishness. I have mine and you don’t.” Some conservatives view a movement toward universal coverage as “a power grab by ‘takers’ whose only motivation is to enjoy a free ride.” (Craig, 1984).

ACA, as first envisioned, supported an expansion of Medicaid financial eligibility in all states. However, opposition to this goal led to opposition and eventual change to permit states to opt out of expansion. Nineteen states initially opted out of Medicaid resulting in a “coverage gap” for many. While there was a nation-wide sharp reduction in the uninsured population, the reduction in the uninsured could have been higher with all states agreeing to the expansion. Those in the coverage gap who remained uninsured most often had income too low to qualify for tax credits but too high to receive Medicaid because their states did not expand financial eligibility (Texas Health Institute, 2016).

Perhaps the barrier to policy change is that some believe in a Social Darwinism approach of survival of the fittest. It is not the function of government to do everything. Instead government should care for those who are strong, with the hope that others through their ambition and with charity can do the rest. Society will benefit if the rich are made richer “and what falls from the table will be enough for the middle class … the wagon train will not make it to the Frontier unless some of the old, some of the young, some of the weak are left behind by the side of the trail.” (Cuomo, 1984).

Perhaps the barrier to policy change is a belief that not all are equal. Consider the transition evident in Orwell’s *Animal Farm,* in its “Seven Commandments” which went from “All animals are equal” to “All Animals are Equal but Some Animals are More Equal than Others.” (Bloom, 2009) Consider disparate access to care and health outcomes in the nation.

Perhaps the barrier to policy change is the belief there are not enough resources for everyone to obtain all the health care that is needed and desired. Bauzon (Bauzon, 2015) asserts it is not possible for everyone to have the right to the best basic care. There is not enough of it to be distributed to everyone. What then should be the ethical and moral bases for rationing? Perhaps the barrier to policy change emanates from a manifestation of an us-versus-them attitude. Related, Pilkington (Pilkington, 2016) employs an us/we-versus the/them approach with regard to medicine. We intimately care for our own health and for those we care about the most. We should be treated congruent with how we would treat ourselves.

## Conclusion

A denial of membership as “one of us,” is antithetical to the foundational values that have been discussed in this paper. If the value of equal justice for all is upheld, health care cannot be divided into haves and have nots. However, the current U.S. health system supports precisely that. There is a health care underclass, in which some are unable to access equal, high quality care, with resultant health status disparities, in conflict with the nation’s democratic values and underlying religious moral and ethical foundations. The rhetoric from the nation’s Presidents and other political leaders are often congruent with these foundations, and yet the current health system is not reflective of these.

The goal of a single payer system provides a pathway to health reform with a values’ foundation. A single payer system permits equal access to the same quality of care, where everyone has “the same card,” and is congruent with the foundational values discussed in this paper.
